# Are Empowered Employees More Proactive? The Contingency of How They Evaluate Their Leader

**DOI:** 10.3389/fpsyg.2017.01802

**Published:** 2017-11-01

**Authors:** Kui Yin, Lu Xing, Can Li, Yungui Guo

**Affiliations:** ^1^Dongling School of Economics and Management, University of Science and Technology Beijing, Beijing, China; ^2^School of Labor and Human Resources, Renmin University of China, Beijing, China; ^3^Guanghua School of Management, Peking University, Beijing, China; ^4^School of Economic and Management, Zhoukou Normal University, Zhoukou, China

**Keywords:** empowering leadership, role breadth self-efficacy, proactive behavior, trust in leader competency, proactive personality

## Abstract

Finding ways to enhance employee proactive behavior is a focal concern for academics and practitioners. Previous studies have found a positive association between empowering leadership and proactive behavior (Martin et al., 2013; Li et al., 2017). However, these studies lack elaboration on mechanisms and do not rule out the effect of employees’ proactive personality during empirical testing. We investigate empowering leadership from individual perspective due to the variation of empowerment levels even in the same team. Our research proposes a more elaborated theoretical model that explains why, and when, empowering leadership might promote employee proactive behavior. Specifically, we examine mediating mechanisms based on social cognitive theory and propose trust in leader competency as boundary condition. Using a sample of 280 leader–follower dyads from a large state-owned Chinese company, our results revealed that (1) empowering leadership was positively related to proactive behavior, with role breadth self-efficacy acting as a mediator for this relationship; (2) employees’ trust in leader competency moderated both the empowering leadership–subordinate proactive behavior relationship and the mediating effect of role breadth self-efficacy, such that the empowering leadership–subordinate proactive behavior relationship was weaker, and the mediating effect of role breadth self-efficacy was stronger, for employees with high levels of trust in leader competency.

## Introduction

In complex and dynamic business environments, organizational effectiveness and competitiveness largely depend on employee proactivity (Parker et al., 2006). There are various manifestations of proactive behaviors, such as feedback seeking, voice, job crafting, taking charge, issue selling, and building social networks (Lam et al., 2014). Proactive behavior has been regarded as an increasingly important component of job performance (Crant, 2000). Previous studies have demonstrated that proactive behaviors are associated with desirable organizational outcomes, such as creativity (Chen and Hou, 2016), task performance (Weseler and Niessen, 2016), job satisfaction (Anseel et al., 2015), and organizational commitment (Saks et al., 2011). Despite the well-documented benefits of proactive behavior, the question of how to promote employee proactivity in the workplace is relatively under-explored (Hong et al., 2016).

Leader behavior is a prominent situational factor in the workplace and is considered a particularly important antecedent of proactive behavior (Belschak and Hartog, 2010). Research has identified that transformational leadership (e.g., Den Hartog and Belschak, 2012), abusive supervision (e.g., Ouyang et al., 2015), and empowering leadership (e.g., Hong et al., 2016) all have an impact on the likelihood that proactive behavior will occur. In this study, we focus on the role of empowering leadership, which is defined as sharing power with their subordinates (Vecchio et al., 2010), with fundamental differences with other leadership styles (Arnold et al., 2000). Compared with traditional hierarchical management styles (e.g., directive leadership), empowering leadership is more closely related to the flexibility and efficiency of today’s organizations, also better in enhancing team performance (Arnold et al., 2000; Lorinkova et al., 2013). With the increasing autonomy for employees (Li et al., 2016), understanding the relationship between empowering leadership and proactive behavior becomes critically significant.

Some researchers have empirically validated that employees’ proactivity increase when supervisors provide empowering leadership (Martin et al., 2013; Hong et al., 2016). However, our understanding of how and when empowering leadership relate to proactive behavior is incomplete based on prior studies. The present study aims to examine the mediating role of role breadth self-efficacy, defined as individuals’ confidence in capability of carrying out a range of activities (Parker, 2000). Role breadth self-efficacy was found to be positively related to individuals’ proactive behavior (Parker et al., 2006; Griffin et al., 2010). To date, however, researchers have not examined the central theoretical role of RBSE might play in explaining the previously identified relationship between empowering leadership and proactive behavior.

Meanwhile, less attention has been paid to the boundary condition of empowering leadership in existing studies. Trust has been extensively examined and conceptualized in management literature (Chughtai et al., 2015). However, most studies focus on the main effect of trust and sparse research investigates the moderating role of trust (Ertürk and Vurgun, 2015). In the present study, we propose that subordinates’ perception of leader trustworthiness, an important contingency of leadership effectiveness (for a review, see Mayer et al., 1995), influences the causal path from empowering leadership to proactive behavior.

Our study provides several important contributions to the extant literature. First, the conceptualizations and measurements of empowering leadership are fragmented across studies (Arnold et al., 2000; Pearce and Sims, 2002; Ahearne et al., 2005; Vecchio et al., 2010; Amundsen and Martinsen, 2014). The most widely used measure of empowering leadership is the four-dimension scale developed by Ahearne et al. (2005), which focused on (1) enhancing the meaningfulness of work, (2) fostering participation in decision making, (3) expressing confidence in high performance, and (4) providing autonomy from bureaucratic constraints. However, Cheong et al. (2016) suggested that the four dimensions can influence proactive behavior through different mechanisms. Our study focuses on the central tenet of empowering leadership, i.e., power-sharing, and adopts a single underlying dimension (Vecchio et al., 2010). In this way, our investigation provides a more consistent logic.

Second, most previous studies do not take employees’ proactive personality into account, with only a few exceptions (e.g., Sonnentag, 2003; Hong et al., 2016). Meanwhile, proactive personality has been found to correlate with proactivity across time and contexts (Bateman and Crant, 1993; Crant, 1995; Parker et al., 2006). Thus, it is often unclear to what extent proactive behavior is endogenous to employees’ proactive personality. We intentionally control for the impact of employee proactive personality, to rule out the alternative explanation, which increases the likelihood that proactive behavior is driven by empowering leadership.

Finally, the current study extends prior research by investigating how and when empowering leadership influences employees’ proactive behavior. Specifically, we test the mediation effect of role breadth self-efficacy and the moderation effect of employee trust in manager’s capabilities to implement work effectively (i.e., competency) (Boyatzis, 1982). Interestingly, we find that employee trust in leader competency weakens the relationship between empowering leadership and proactive behavior, while strengthening the relationship between empowering leadership and self-efficacy.

## Theoretical Background and Hypotheses Development

### Empowering Leadership

The widely adopted definition of empowering leadership is described by Ahearne et al. (2005) as the degree of manifestation of four leadership behaviors: “enhancing the meaningfulness of work, fostering participation in decision making, expressing confidence in high performance, and providing autonomy from bureaucratic constraints.” However, the four-dimensional definition of empowering leadership conceptually overlaps with other related constructs, such as transformative leadership (Derue et al., 2011) and leader–member exchange (Hassan et al., 2013), which, to some extent, results in vagueness when explicating the functional mechanism of the model (Amundsen and Martinsen, 2014). Therefore, in the present study, we define empowering leadership as a single-dimensional construct, whereby power is shared with subordinates (Vecchio et al., 2010). Hong et al. (2016) also adopted Vecchio et al.’s definition of empowering leadership. The notion of empowerment becomes important because it enables employees to be effective (Spreitzer, 1995). Correspondingly, the impact of empowering leadership on positive outcomes for employees has been extensively justified to date, such as task motivation/psychological adaptation (Amundsen and Martinsen, 2014), employee creativity (Zhang and Zhou, 2014), service performance (Wu and Chen, 2015), job satisfaction, organizational commitment, and organizational citizenship behavior (Fong and Snape, 2015).

In this study, we emphasized perceived empowering leadership. Empowering leadership can be conceptualized at both the team level and individual level (Fong and Snape, 2015; Li and Zhang, 2016). At the team level, there is an assumption that leader behavior may be perceived as invariant by subordinates. However, increasingly scholars realize that leaders empower subordinates differently, depending on subordinates’ abilities, attitudes, and the quality of leader–member exchange. For example, Vecchio et al. (2010) found that empowering leadership is positively associated with higher job satisfaction and leader-rated performance, based on superior–subordinate dyads data. Several studies have emphasized that leaders should treat each employee differently, which is consistent with situational leadership theory (Vecchio et al., 2010; Amundsen and Martinsen, 2014). In this study, we adopt an individual perspective and measure empowering leadership in the superior–subordinate dyadic situation, following the approach taken by Vecchio et al. (2010).

### Empowering Leadership and Proactive Behavior

Proactive behavior has received considerable attention from academics (Dysvik et al., 2016). Proactive behavior also referred to as personal initiative or proactivity, is a specific form of work motivation. It can be defined as “self-starting, anticipatory, long-term oriented and persistent work behaviors of individual employees” (Frese and Fay, 2001). Examples of proactive behavior include: seizing opportunities, predicting and preventing risks, and advancing and improving the current circumstances (Parker et al., 2010). Several lines of empirical research support the idea that proactive behavior promotes positive work-related outcomes, such as job performance, career satisfaction, and employment opportunities (Parker et al., 2010). Bolino and Turnley (2005) distinguish between the construct of proactive behavior and organizational citizenship behavior, in that the definition of the former is broader and covers both in-role and extra-role behaviors. Empowering leadership, as a contextual factor, can influence employees’ proactive behavior along with proactive personality (Parker et al., 2010). Recent theorizations by Parker et al. (2010) support a more comprehensive view of proactive behavior, by integrating both individual and contextual factors. A leader is one of the most important contextual factors in the workplace (Chen et al., 2002).

Prior research has indicated that proactive behaviors, such as change-oriented organizational citizenship behavior (Li et al., 2016), are more likely to occur when employees feel empowered by their leaders. Empowering leadership involves granting employees a fair amount of autonomy, which consists of sharing power with subordinates and authorizing employees to do their jobs on their own (Vecchio et al., 2010). Employees with autonomy are more likely to feel responsibility for their jobs and be motivated to conduct change-oriented behaviors (Parker et al., 2006). In a field experiment, Martin et al. (2013) demonstrated that empowering leadership, rather than directive leadership, increased proactive behaviors. Proactive personality is the most important individual factor in predicting proactive behavior (Thomas et al., 2010). Empowering leadership and proactive personality play different roles in encouraging proactive behaviors. Empowering leaders provide information that employee themselves are able to behave proactively. Proactive personality gives employees themselves the intrinsic motivation to behave in a proactive way (Parker et al., 2010).

Despite the importance of proactive personality, empowering leadership also accounts for variances in proactive behavior. In other words, empowering leadership, as a contextual factor, can influence employees’ proactive behavior in addition to proactive personality. Hong et al. (2016) found that empowering leadership has a cross-level influence on proactive behavior by fostering a proactive atmosphere, after controlling for proactive personality. Taking these findings together, we hypothesize as follows:

**Hypothesis 1:** Empowering leadership is positively related to proactive behaviors.

### The Mediating Role of Role Breadth Self-efficacy

We focus on role breadth self-efficacy because performing proactive behavior involves engaging in tasks that go beyond prescribed requirements. Role breadth self-efficacy refers to one’s perceived capability to perform a range of interpersonal, proactive, and integrative activities (Parker et al., 2006). Role breadth self-efficacy has been shown to be associated with positive outcomes, such as innovative performance (e.g., Chen et al., 2013), perceived employability (e.g., Kim et al., 2015), and proactive behavior (Lee et al., 2016).

To begin with, we expect a positive effect of empowering leadership on role breadth self-efficacy. According to social cognitive theory, individual self-efficacy is malleable and can be developed. People form efficacy beliefs mainly through mastery experience, vicarious learning, and persuasive words. Empowering leaders can cultivate employees’ self-efficacy. First, employees receive cues as to what is rewarded and expected in the organization by interacting with their immediate leader (Bowen and Ostroff, 2004). Empowering leaders encourage followers to take on responsibilities and collaborate with others to handle problems (Vecchio et al., 2010), conveying the message that employees can do jobs on their own. Second, empowering leadership offers employees support for pursuing unstructured tasks (Martin et al., 2013). Employees have more opportunities to perform various tasks and accumulate mastery experience. Jonsson et al. (2015) found that there was a direct relationship between empowering leadership and learning (both in Swedish and Chinese samples). An empirical study from China also proved that empowering leadership positively affects followers’ role breadth self-efficacy (Li et al., 2015). Thus, we expect that empowering leadership will be positively related to role breadth self-efficacy.

Role breadth self-efficacy enhances proactive behavior (López-Domínguez et al., 2013). Employees with high role breadth self-efficacy perceive their job roles more broadly and conduct a wider range of tasks than employees with lower role breadth self-efficacy (Kim et al., 2015). Given that proactive action can incur risks and uncertainty, it is important for employees to have a strong belief that they can bring about change and cope with potential obstacles. Individuals with high role breadth self-efficacy see opportunities in their environment and perceive an increased likelihood of success through proactive behavior (Wu and Parker, 2017). Extant research reports that role breadth self-efficacy is a strong predictor of behaviors, such as change-oriented organizational citizenship behavior (e.g., López-Domínguez et al., 2013) and proactive behavior (e.g., Parker et al., 2006). Work by Hwang et al. (2015) has suggested that role breadth self-efficacy is positively related to both pro-organizational proactive behaviors and dyad-referenced interpersonal proactive behaviors. Parker et al. (2010) posited that proactive behavior is a “motivated, conscious, and goal directed” process driven by “can do” (expectancy), “reason to,” and “energized to” attitudes. Using Parker et al.’s framework, Hong et al. (2016) found that only “can do” attitudes presented by role breadth self-efficacy influenced proactive behavior, while intrinsic motivation and activated positive affect had no significant impact. In light of these findings, we propose that role breadth self-efficacy mediates the relationship between empowering leadership and proactive behavior.

**Hypothesis 2:** Role breadth self-efficacy mediates the relationship between empowering leadership and proactive behavior.

### The Moderating Role of Trust in Leader Competency

It is widely acknowledged that trust is a critical element and facilitator of organizational success (Dirks and Ferrin, 2001). Nyhan and Marlowe (1997) distinguished between trust at the interpersonal and organizational level. Meanwhile, trust has been identified as an important aspect of leadership theories (Ötken and Cenkci, 2012). Trust in supervisors is shown to be positively related to leader–member exchange (e.g., Chen et al., 2012), employees’ work-related well-being (e.g., Chughtai et al., 2015), and perceived interactional justice (e.g., Wu et al., 2012). In other words, employees’ trust in their leader is a key predictor of leadership effectiveness (Burke et al., 2007). The underlying rationale is that employees who view the leader as capable and competent are more likely to accept their authority (Spector, 1985; Martin et al., 2013). Therefore, in the context of this study, we focus on trust in leader competence as a boundary condition for the predicted relationship between empowering leadership and both role breadth self-efficacy and proactive behavior. Trust in leader competency reflects employees’ own perceptions and feelings that the supervisor is competent in his or her job (Nyhan and Marlowe, 1997).

First of all, when empowering leadership is implemented, we expect that role breadth self-efficacy increases for those employees with higher levels of trust in leader competency. Corresponding to the explanation of the mediation effect, we adopt two theoretical perspectives to analyze the moderation effect of trust in leader competency. First, managers are salient information sources in a workplace context and play a vital role in shaping employees’ perceptions and attitudes (Nishii and Wright, 2008). Under empowering leadership, employees are delegated power to take additional responsibilities and given decision-making authority (Ahearne et al., 2005). According to attribution theory, employees will try to interpret and identify the reasons for such leader behaviors. The empowering leadership approach can be interpreted as either beneficial or harmful, depending on employees’ earlier priming (Fiske and Taylor, 1984). When trust in leader competency is high, employees are confident that they will engage in a leadership approach that will ultimately prove to be beneficial (Ötken and Cenkci, 2012). Employees are more likely to be vulnerable to their leaders under this circumstance (Mayer and Gavin, 2005). As a result, employees tend to attach more importance to the informational cues provided by leaders. Specifically, as we illustrated above, the persuasive messages from empowering leadership are more salient. Accordingly, we anticipate that high trust in leader competency enables employees to experience a stronger role breadth self-efficacy under empowering leadership. On the contrary, a low level of trust in leader competency might lead subordinates to suspect that empowering leadership is a way for leaders to shift responsibility, therefore refusing to accept the influence of their leaders. In this situation, empowering leadership does not necessarily facilitate role breadth self-efficacy.

Second, when subordinates believe that their leaders are competent in the job – for instance, when they are following through on assignments, making well-thought-out decisions, doing jobs in an acceptable manner, and avoiding causing other problems (Nyhan and Marlowe, 1997) – employees will build up a sense of security and attachment to their leaders’ managerial practices (Ertürk and Vurgun, 2015). In such instances, subordinates will be more willing to share information and cooperate with their leader (Chen et al., 2012). As discussed earlier, empowering leadership enhances followers’ role breadth self-efficacy through enactive mastery experience, according to the perspective of social learning theory (Bandura, 1977). With a high level of trust in leader competency, employees tend to reciprocate and are more willing to carry out tasks and strategies (Burke et al., 2007). Subsequently, the possibility to derive mastery experience from daily work will increase. Therefore, we can infer that trust further facilitates the positive effect of empowering leadership on role breadth self-efficacy. On the contrary, employees with an absence of trust in their leaders’ competency transfer work experience to self-efficacy with less effectiveness. The positive effect of empowering leadership on role breadth self-efficacy may dwindle.

Taking these arguments together, we hypothesize a strengthened relationship between empowering leadership and role breadth self-efficacy for those employees who view their leaders as competent, whereas we predict a weakened relationship for employees with low levels of trust in their leader’s competency.

**Hypothesis 3:** Trust in leader competency will positively moderate the impact of empowering leadership on role breadth self-efficacy. Empowering leadership has a stronger positive effect on role breadth self-efficacy when trust in leader competency is higher rather than lower.

Assuming trust in leader competency moderates the association between empowering leadership and role breadth self-efficacy, it is also likely that trust in leader competency will conditionally influence the strength of the indirect relationship between empowering leadership and proactive behavior, thereby demonstrating a pattern of moderated mediation between the focal variables. Because we predict a strong (weak) relationship between empowering leadership and role breadth self-efficacy when trust in leader competency is high (low), we expect the following:

**Hypothesis 4:** Trust in leader competency will positively moderate the indirect relationship between empowering leadership and proactive behavior via role breadth self-efficacy, such that the indirect link will be stronger when leader competency is higher rather than lower.

Besides the indirect effect through role breadth self-efficacy, employees also exert proactive behavior based on direct experience of empowering leadership. Our study also investigates the role of trust in leader competency in moderating the direct relationship between empowering leadership and proactive behavior. Contrary to intuition, we propose that trust in leader competency negatively moderates this direct effect. First, when employees do not trust their leaders to perform well, they are more likely to prefer the autonomy associated with empowering leadership. Kramer (1999) found that trust engenders sociability, increasing employees’ willingness to communicate. On the contrary, employees with less trust in their leaders tend to reduce interaction with their leaders. For those employees with less trust in leader competency, empowerment provided by supervisors offer additional autonomy, which is in consistence with employees’ preferences and associated with positive affect (Martin et al., 2013). Positive affect further promotes proactive behavior (Fritz and Sonnentag, 2009). Second, employees with low trust in leader competency are less likely to identify with their leaders (Tjosvold, 1984). As a result, when employees view their leaders as incompetent, they would prefer to make their own decisions. This is exactly what empowering leadership encourages (Vecchio et al., 2010). Therefore, employees with lower level of trust in leader competency are more likely to seize the opportunities to exert more proactive behaviors.

Those arguments are consistent with the findings of Martin et al. (2013), who found that work units that had lower satisfaction with their leaders, prior to the implementation of empowering leadership, experienced greater improvement in proactivity than work units that were more satisfied with their leaders. Thus, employees with high levels of trust in leader competency are more likely to forge a weak link between empowering leadership and proactive behavior. On the contrary, competent leaders tend to be charismatic in the eyes of employees who trust their leaders’ competency, which enhances the subordinates’ followership. As a result, employees with high levels of trust in leader competency are less likely to value autonomy in performing job tasks, which consequently weakens the direct association between empowering leadership and proactive behavior. Taking these findings together, we hypothesize as follows:

**Hypothesis 5:** Trust in leader competency will negatively moderate the direct relationship between empowering leadership and proactive behavior, such that the link between empowering leadership and proactive behavior will be weaker when leader competency is higher rather than lower.

## Materials and Methods

### Participants and Procedure

Our data were collected from a large state-owned company in China through printed surveys. China was especially suitable for our empirical setting, because it is a country with a high power distance culture, where employees’ work behaviors are significantly influenced by their leaders. With the consent of the person in charge of the company, we got the list of all staff names including leaders and subordinates, which had presented who is the superior to each subordinates. Then we randomly sampled 400 subordinates. According to the list of names, we marked each subordinate’s questionnaire in order to let us know who fills out the questionnaire, which was only known by researchers to ensure privacy of the participants. Human resource manager of the company helped us distribute the marked questionnaires to corresponding subordinates. In the meantime, we invited the drawn subordinates’ leaders to evaluate their subordinates in terms of proactive behavior. Data on employees’ demographics (e.g., age, gender, education, work tenure, and dyadic tenure), empowering leadership, role breadth self-efficacy, leader competence trust, and proactive personality were collected from subordinates, whereas data on proactive behavior were gathered from their leaders.

We distributed 400 questionnaires to subordinates and received 325 valid questionnaires, a response rate of 81.25%. We distributed 88 questionnaires to the supervisors of the 400 employees and received 75 usable questionnaires from 80 supervisors, a response rate of 85.23%. After matching the supervisors and subordinates, the final sample consisted of 280 employees and 72 supervisors. For the employees, 55.23% were female. The average age of employees participating in this study was 33.88 years (*SD* = 7.84), ranging from 20 to 58 years old. Regarding education, 2.15% finished high school, 16.13% held junior college degrees, 55.20% held bachelor degrees, and 26.52% held master degrees or higher. Average work tenure was 9.57 years (*SD* = 8.9) and average dyadic tenure was 4.01 (*SD* = 4.16).

### Ethics Statement

An ethics approval was not required as per institutional guidelines and national laws and regulations because no unethical behaviors existed in the research process. We just conducted paper-pencil test and were exempt from further ethics board approval since our study did not involve human clinical trials or animal experiments. In the first page of the questionnaire, we informed participants about the objectives of the study and guaranteed their confidentiality and anonymity. They were completely free to join or drop out the survey. Only those who were willing to participate were recruited.

### Measures

Except for the empowering leadership scale (Vecchio et al., 2010), other scales used in this study have been validated in prior studies conducted in China. We created a Chinese version of the empowering leadership scale, following the translation–back translation procedure (Brislin, 1970).

#### Empowering Leadership

A 10-item scale developed by Vecchio et al. (2010) was used to measure empowering leadership, which was validated by Hong et al. (2016). Sample items included “My supervisor encourages me to find solutions to my problems without his/her direct input.” Participants were asked to indicate on a 6-point Likert-type scale, ranging from 1 (*completely disagree*) to 6 (*completely agree*). In the current study, Cronbach’s alpha was 0.91.

#### Role Breadth Self-efficacy

Role breadth self-efficacy was measured using 7 items developed by Parker et al. (2006), which was validated by Fuller et al. (2012) and Hong et al. (2016). Sample items include “How confident would you feel about representing your work area in meetings with senior management.” The rating scale was anchored at 1 (*not at all confident*) and 6 (*very confident*). In the current study, Cronbach’s alpha was 0.92.

#### Proactive Behavior

We measured proactive behavior using 6 items developed by Fuller et al. (2012). Li and Tian (2014) used this scale in their study in China. A sample item was: “This person often tries to bring about improved procedures for the work unit or department.” Participants were asked to rate on a 6-point Likert-type scale, ranging from 1 (*completely disagree*) to 6 (*completely agree*). In the current study, Cronbach’s alpha was 0.88.

#### Trust in Leader Competency

Trust in leader competency was measured using 6 items developed by Nyhan and Marlowe (1997), which has been validated in the Chinese context (He, 2010). Sample items include: “My supervisor is technically competent at the critical elements of his or her job,” and “My supervisor is able to do his or her job in an acceptable manner.” Participants were asked to indicate on a 6-point Likert-type scale, ranging from 1 (*completely disagree*) to 6 (*completely agree*). In the current study, Cronbach’s alpha was 0.95.

#### Control Variables

We considered several control variables. Referring to previous studies (e.g., Fuller et al., 2012; Hong et al., 2016), we controlled for age (years), gender (dummy-coded such that male = 0), education (1 = high school, 2 = junior college, 3 = bachelor, 4 = master), work tenure (years), dyadic tenure (years), and proactive personality at the employee level. We measured proactive personality using 10 items developed by Seibert et al. (1999), on a scale from 1 (*completely disagree*) to 6 (*completely agree*). Sample items include: “I am always looking for a better way of doing things” and “Wherever I have been, I have been a powerful force for constructive change.”

### Statistical Analysis

We used SPSS22 to conduct descriptive analysis, correlation analysis, and reliability analysis. Confirmatory factor analysis was also conducted to verify the distinctive validity among current variables. Because our model involved mediation, moderation, and moderated mediation in the same time, we adopted Mplus7 to examine the moderated mediating model through path analysis as recommended by prior studies. Following the recommendations of Preacher et al. (2010), we tested all hypotheses simultaneously, rather than using the causal steps approach. In order to test Hypothesis 2 and Hypothesis 4, we examined the indirect effects of empowering leadership on employees’ proactive behavior via role breadth self-efficacy, with the parametric bootstrap method using Mplus7. To date, bootstrap methods are preferred over normal distribution-based significance tests (Preacher et al., 2010).

## Results

### Descriptives and Correlations among Variables

We computed means, standard deviations, and zero-order correlations among the variables and controls in this study, as shown in **Table 1**. The results show that all relationships point in the expected direction and correlation coefficients are not larger than 0.6 (except for the link between proactive personality and role breadth self-efficacy), which implies that there is good discrimination validity among the main variables. Specifically, empowering leadership is positively related to role breadth self-efficacy (*r* = 0.31, *p* < 0.01), trust in leader competency (*r* = 0.50, *p* < 0.01), and proactive behavior (*r* = 0.31, *p* < 0.01). There is a significant relationship between role breadth self-efficacy and proactive behavior (*r* = 0.28, *p* < 0.01).

**Table 1:** Descriptive statistics and bivariate correlations among variables in the study (*N* = 280).

Variables	*M*	*SD*	1	2	3	4	5	6	7	8	9	10
(1) Gender	0.55	0.50										
(2) Age	33.88	7.84	0.00									
(3) Education	3.07	0.73	0.02	-0.38**								
(4) Work tenure	9.57	8.90	-0.05	0.78**	-0.41**							
(5) Dyadic tenure	4.01	4.16	0.03	0.36**	-0.23**	0.48**						
(6) Proactive personality	4.37	0.83	-0.05	0.02	0.15*	0.06	0.04	**0.88**				
(7) Empowering leadership	4.31	0.88	0.03	-0.12*	0.11	-0.05	-0.04	0.33**	**0.91**			
(8) Role breadth self-efficacy	4.35	1.08	-0.10	0.10	0.12*	0.10	0.01	0.62**	0.31**	**0.92**		
(9) Trust in leader competency	5.08	1.03	0.11	-0.13*	0.20**	-0.15*	-0.01	0.24**	0.50**	0.22**	**0.95**	
(10) Proactive behavior	3.97	0.88	-0.04	0.05	0.00	0.15*	0.17**	0.19**	0.23**	0.28**	0.17**	**0.88**

*Bold figures on the diagonals are scale reliabilities (Cronbach’s alpha). ^∗^*p* < 0.05, ^∗∗^*p* < 0.01*.

### Testing the Hypotheses

**Table 2** reports the coefficients of path analysis used to test our hypotheses. Our results suggest a significant relationship between empowering leadership and proactive behavior (*b* = 0.26, *p* < 0.05), after controlling for employees’ gender, age, education, work tenure, dyadic tenure, and proactive personality. This finding provides support for Hypothesis 1. We posited that role breadth self-efficacy mediates the relationship between empowering leadership and proactive behavior. Empowering leadership predicts role breadth self-efficacy (*b* = 0.29, *p* < 0.01) and role breadth self-efficacy influences proactive behavior (*b* = 0.20, *p* < 0.01), which supports the mediating role of role breadth self-efficacy. To obtain a steadier estimate, we conducted bias-corrected bootstrap analysis using 2000 bootstrap samples. **Table 3** displays the results of our analyses. The 95% confidence interval of the mediating effect is [0.02, 0.11] and does not include zero. Thus, Hypothesis 2 is supported.

**Table 2:** Results of path analysis (*N* = 280).

	Role breadth self-efficacy			Proactive behavior		
	*b*	*SE*	95% IC	*b*	*SE*	95% IC
Intercept	-0.06**	0.08	[-0.24, 0.08]	4.18**	0.06	[4.08, 4.30]
Gender	0.05*	0.18	[-0.47, 0.12]	0.00	0.07	[-0.10, 0.22]
Age	0.03	0.01	[0.01, 0.06]	-0.01	0.01	[-0.04, 0.20]
Education	0.08	0.07	[-0.09, 0.19]	0.03	0.05	[-0.05, 0.03]
Work tenure	0.01	0.01	[-0.02, 0.03]	0.01	0.01	[-0.01, 0.07]
Dyadic tenure	-0.01	0.02	[-0.06, 0.03]	0.04*	0.01	[0.01, -0.05]
Proactive personality	-0.04	0.13	[-0.13, 0.26]	-0.11	0.07	[-0.28, -0.05]
Empowering leadership	0.29**	0.09	[0.13, 0.48]	0.26**	0.07	[0.13, 0.39]
Role breadth self-efficacy				0.20**	0.06	[0.08, 0.31]
Trust in leader competency	0.21*	0.11	[0.03, 0.43]	-0.11	0.07	[-0.25, 0.01]
Empowering leadership ^∗^ Trust in leader competency	0.14*	0.07	[0.02, 0.28]	-0.15**	0.06	[-0.26, -0.03]

^∗^*p < 0.05, ^∗∗^*p* < 0.01*.

**Table 3:** Path analysis results to test moderated mediation model (*N* = 280).

	Empowering leadership → role breadth self-efficacy → proactive behavior			
Indirect effect	0.06 [0.02, 0.11]			
Group summary	First stage	Indirect effect	Direct effect	Total effect
High trust in leader competency (+1 *SD*)	0.43 [0.21, 0.68]	0.09 [0.04, 0.16]	0.11 [-0.06, 0.28]	0.20 [0.02, 0.37]
Low trust in leader competency (-1 *SD*)	0.15 [-0.06, 0.34]	0.03 [-0.01, 0.09]	0.41 [0.24, 0.61]	0.44 [0.25, 0.63]
Intergroup difference	0.28 [0.04, 0.58]	0.06 [0.01, 0.13]	-0.30 [-0.51, -0.06]	-0.24 [-0.47, 0.02]

^∗^*p < 0.05, ^∗∗^*p* < 0.01*.

As predicted, trust in leader competency positively moderated the relationship between empowering leadership and role breadth self-efficacy (*b* = 0.14, *p* < 0.05, **Table 2**). Therefore, Hypothesis 3 is supported. In addition, our results also revealed that trust in leader competency negatively moderated the direct effect of empowering leadership on proactive behavior (*b* = -0.15, *p* < 0.05, see **Table 2**), thus, Hypothesis 5 is supported.

As indicated in **Table 3**, the conditional indirect effect of empowering leadership on proactive behavior via role breadth self-efficacy was significant when trust in leader competency was high (*b* = 0.09, 95% CI [0.04, 0.16]), but insignificant when trust in leader competency was low (*b* = 0.03, *ns*). These results support Hypothesis 4 and reveal that trust in leader competency strengthens the mediating effect of role breadth self-efficacy in the relationship between empowering leadership and proactive behavior.

**FIGURE 1:**
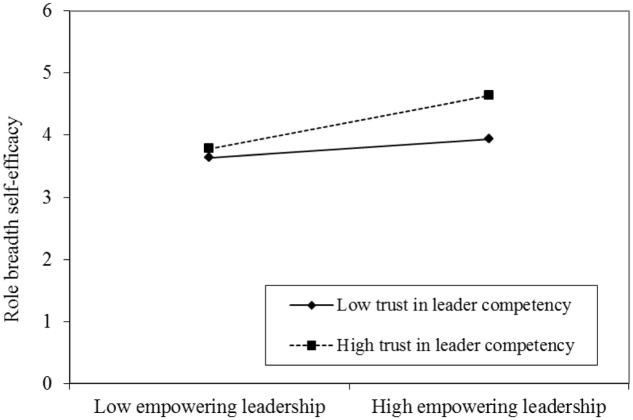
Moderating effect of trust in leader competency on the relationship between empowering leadership and role breadth self-efficacy.

To better interpret the interaction patterns, we plotted the simple slope at one standard deviation above and below the mean of trust in leader competency (see **Figure 1**). When trust in leader competency is high, the link between empowering leadership and role breadth self-efficacy is stronger (*b* = 0.43, *p* < 0.01, see **Table 3** and **Figure 1**). On the contrary, the relationship is not significant (*b* = 0.15, *ns*, see **Table 3** and **Figure 1**).

**FIGURE 2:**
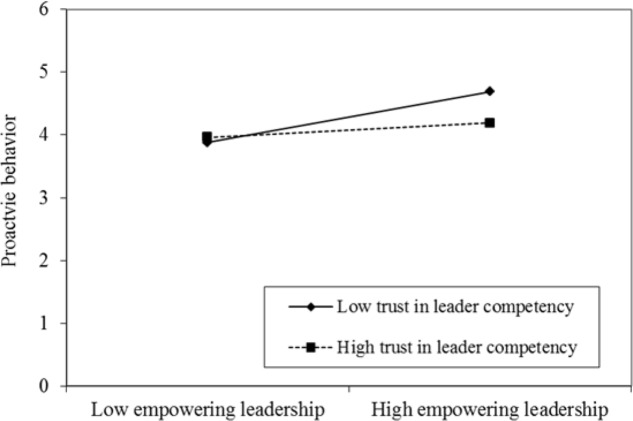
Moderating effect of trust in leader competency on the relationship between empowering leadership and proactive behavior.

To better interpret the interaction patterns, we plotted the simple slope at one standard deviation above and below the mean of trust in leader competency (see **Figure 2**). When trust in leader competency is high, the link between empowering leadership and proactive behavior is weakened (*b* = 0.11, *ns*, see **Table 3** and **Figure 2**). On the contrary, the relationship is stronger (*b* = 0.41, *p* < 0.01, see **Table 3** and **Figure 2**).

**FIGURE 3:**
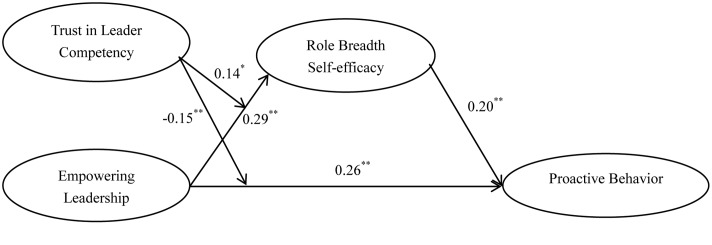
Results of research model. ^∗^*p* < 0.05, ^∗∗^*p* < 0.01.

In order to better illustrate the results, **Figure 3** listed all path coefficients of the whole model. All the relationship directions among variables are in consistent with our assumptions.

## Discussion

To promote employee proactive behavior, this study constructed a framework that combined empowering leadership, role breadth self-efficacy, proactive behavior, and trust in leader competency. We developed a moderated mediating model to examine how empowering leadership shapes employees’ proactive behavior. After controlling for proactive personality, our results showed that perceived empowering leadership was positively related to proactive behavior. In addition, role breadth self-efficacy mediated the relationship between empowering leadership and proactive behavior. Interestingly, trust in leader competency played a different moderating role in the direct and indirect effect of empowering leadership on proactive behavior.

Our study has several important implications for research on proactive behavior. First, there is a lack of research to control personality in exploring the effect of empowering leadership on proactive behavior. We shed light on the net effect of empowering leadership on proactive behavior through including proactive personality as a control variable at the individual level. Although extant studies have demonstrated that empowering leadership increases proactive behavior (e.g., Li et al., 2015, 2016, 2017), these studies did not control for proactive personality methodologically, and thus cannot distinguish between the contextual effect of empowering leadership and the individual effect of proactive personality. Hong et al. (2016) controlled for proactive personality when analyzing the process in which empowering leadership influences proactive behavior at the departmental level, but they did not test the direct effect of empowering leadership. Our study shows that empowering leadership has a positive effect on employees’ proactive behavior at the individual level, in a more rigorous manner.

Second, we found that role breadth self-efficacy meditates the relationship between empowering leadership and proactive behavior. Our results provide encouraging empirical evidence for Parker et al.’s (2010) model, demonstrating that distal contextual variables (e.g., empowering leadership) shape proximal motivational states (e.g., role breadth self-efficacy), which further shapes proactive behavior. Our results also confirm those of previous studies, which concluded that role breadth self-efficacy is an important mechanism by which empowering leadership plays a pivotal role (Parker et al., 2006; Martin et al., 2013; Hong et al., 2016). Particularly, we explicitly delineate that the core component of empowering leadership, sharing power with subordinates, enhances employees’ role breadth self-efficacy.

Third, our findings on the moderating effects of trust in leader competency heed the call for research on the boundary conditions of empowering leadership (Sharma and Kirkman, 2015). We found that trust in leader competency negatively moderates the relationship between empowering leadership and proactive behavior. This is consistent with substitute for leadership theory, in that trust in leader competency substitutes for the effect of leaders’ empowering behavior. In a similar vein, Martin et al. (2013) showed that work units with lower satisfaction with their leaders experienced greater improvement in proactivity, due to the implementation of empowering leadership. On the contrary, trust in leader competency positively moderates the link between empowering leadership and role breadth self-efficacy. The positive moderation effect supports the argument that trust in supervisor competency affects attribution and preference of leader behavior (e.g., Ertürk and Vurgun, 2015).

Our findings also have implications for management. First, given the fact that empowering leadership promotes employee proactive behaviors, leaders should adopt a series of empowering behaviors, such as advising their subordinates to search for solutions initiatively, encouraging their subordinate to look for opportunities in problem-solving processes, urging their subordinates to work as a team with the other employees who work at the organization, and etc. (Vecchio et al., 2010). In addition, organizations can train leaders on how to empower employees effectively (Li et al., 2016). Alternatively, organizations can selectively recruit and promote candidates who own higher tendency to empower to management positions. Second, role breadth self-efficacy is an important motivational state that promotes proactive behavior. On the one hand, high role breadth self-efficacy can be used as an important selection criterion in the process of recruiting. On the other hand, organizations should try to foster an organizational culture that encourages autonomy, job rotation, and information sharing. Third, our moderator analyses suggest that trust in leader competency plays the opposite role in the direct and indirect effect of empowering leadership, which might seem confusing. We cautiously advise that leaders with low competency should empower their subordinates to reach their potential.

A few limitations of this study should be noted. First, due to the cross-sectional research design, we cannot rule out the possibility of alternated ordering of our variables. We relied on employees to report on empowering leadership. Future studies can attempt to investigate team-level empowering leadership. Second, combining the positive and negative moderating effects of trust in leader competency, our results show that the total effect of empowering leadership on proactive behavior was only marginally significant. As **Table 3** showed, the intergroup difference of total effect was not significant (95% CI [–0.47, 0.02]), but marginally significant (90% CI [–0.43, –0.04]). Third, our empirical studying was situated in China’s context. Researchers should be cautious in generalizing our findings in other national context. Future studies may utilize a larger and multinational sample to validate the moderating effect of trust in leader competency for the total effect of empowering leadership on proactive behavior.

Our findings also raise several interesting questions that warrant further exploration. Although we found that empowering leadership contributed to employees’ proactive behavior, after controlling for proactive personality, there is still the question of whether the effects of empowering leadership are greater than that of proactive behavior. As Parker et al. (2010) suggest, proactive behavior is influenced by both contextual factors and individual differences. To our knowledge, no existing theories have been developed to determine which type of predictor is most prevalent. In the future, meta-analysis would be a useful way to summarize the predictor of proactive behavior and conduct multi-group comparisons. There are additional questions concerning the measurement of empowering leadership, such as whether it makes a difference which measurements are selected to predict outcomes. Different empowering leadership measurements include different dimensions. To date, no empirical study has examined the predictive differences of the above measures. Thus, further research is necessary to examine whether the findings reported here replicate with different measurements. Further, we note that future research should attempt to identify other boundary conditions in which empowering leadership affects proactive behavior, such as job control, procedural justice, job stressors (Parker et al., 2010; Sharma and Kirkman, 2015), and employees’ competency. Future research can further explore the effect of competency fit between leader and employees on the effectiveness of empowering leadership.

## Conclusion

In this study, we have extended the literature on proactive behavior by elaborating the link between empowering leadership and proactive behavior. Our results show that empowering leadership has a positive effect on proactive behavior, even after controlling for employees’ proactive personality. In particular, our results suggest that empowering leadership is associated with role breadth self-efficacy – proactive behavior and role breadth self-efficacy mediates the relationship between empowering leadership and proactive behavior. Furthermore, our results suggest a moderated mediated model, in that trust in leader competency both moderates the direct and indirect effect of empowering leadership, via role breadth self-efficacy, in opposite directions.

## Author Contributions

All participated in the study design. KY, LX, and CL wrote the first draft. YG conducted the data analysis and wrote the results section.

## Conflict of Interest Statement

The authors declare that the research was conducted in the absence of any commercial or financial relationships that could be construed as a potential conflict of interest.

## References

[B1] Ahearne M. Mathieu J. Rapp A. (2005). To empower or not to empower your sales force? An empirical examination of the influence of leadership empowerment behavior on customer satisfaction and performance. *J. Appl. Psychol.* 90 945–955. 10.1037/0021-9010.90.5.945 16162066

[B2] Amundsen S. Martinsen ØL. (2014). Empowering leadership: construct clarification, conceptualization, and validation of a new scale. *Leadersh. Q* 25 487–511. 10.1016/j.leaqua.2013.11.009

[B3] Anseel F. Beatty A. S. Shen W. Lievens F. Sackett P. R. (2015). How are we doing after 30 years? A meta-analytic review of the antecedents and outcomes of feedback-seeking behavior. *J. Manage.* 41 318–348. 10.1177/0149206313484521

[B4] Arnold J. A. Arad S. Rhoades J. A. Drasgow F. (2000). The empowering leadership questionnaire: the construction and validation of a new scale for measuring leader behaviors. *J. Organ. Behav.* 21 249–269.

[B5] Bandura A. (1977). *Social Learning Theory.* Englewood Cliffs, NJ: Prentice-Hall.

[B6] Bateman T. S. Crant J. M. (1993). The proactive component of organizational behavior: a measure and correlates. *J. Organ. Behav.* 14 103–118. 10.1002/job.4030140202

[B7] Belschak F. D. Hartog D. D. N. (2010). Pro-self, prosocial, and pro-organizational foci of proactive behaviour: differential antecedents and consequences. *J. Occup. Organ. Psychol.* 83 475–498. 10.1348/096317909X439208

[B8] Bolino M. C. Turnley W. H. (2005). The personal costs of citizenship behavior: the relationship between individual initiative and role overload, job stress, and work-family conflict. *J. Appl. Psychol.* 90 740–748. 10.1037/0021-9010.90.4.740 16060790

[B9] Bowen D. E. Ostroff C. (2004). Understanding HRM-firm performance linkages: the role of the “strength” of the HRM system. *Acad. Manage. Rev.* 29 203–221. 10.2307/20159029

[B10] Boyatzis R. E. (1982). *The Competent Manager: A Model for Effective Performance.* New York, NY: Wiley-Interscience.

[B11] Brislin R. W. (1970). Back-translation for cross-cultural research. *J. Cross Cult. Psychol.* 1 185–216. 10.1177/135910457000100301

[B12] Burke C. S. Sims D. E. Lazzara E. H. Salas E. (2007). Trust in leadership: a multi-level review and integration. *Leadersh. Q.* 18 606–632. 10.1016/j.leaqua.2007.09.006

[B13] Chen A. S. Hou Y. (2016). The effects of ethical leadership, voice behavior and climates for innovation on creativity: a moderated mediation examination. *Leadersh. Q.* 27 1–13. 10.1016/j.leaqua.2015.10.007

[B14] Chen G. Farh J. L. Campbell-Bush E. M. Wu Z. Wu X. (2013). Teams as innovative systems: multilevel motivational antecedents of innovation in R&D teams. *J. Appl. Psychol.* 98 1018–1027. 10.1037/a0032663 23565898

[B15] Chen Z. Lam W. Zhong J. A. (2012). Effects of perceptions on LMX and work performance: effects of supervisors’ perception of subordinates’ emotional intelligence and subordinates’ perception of trust in the supervisor on LMX and, consequently, performance. *Asia. Pac. J. Manage.* 29 597–616. 10.1007/s10490-010-9210-z

[B16] Chen Z. X. Tsui A. S. Farh J. L. (2002). Loyalty to supervisor vs. organizational commitment: relationships to employee performance in china. *J. Occup. Organ. Psychol.* 75 339–356. 10.1348/096317902320369749

[B17] Cheong M. Spain S. M. Yammarino F. J. Yun S. (2016). Two faces of empowering leadership: enabling and burdening. *Leadersh. Q.* 27 602–616. 10.1016/j.leaqua.2016.01.006

[B18] Chughtai A. Byrne M. Flood B. (2015). Linking ethical leadership to employee well-being: the role of trust in supervisor. *J. Bus. Ethics* 128 653–663. 10.1007/s10551-014-2126-7

[B19] Crant J. M. (1995). The proactive personality scale and objective job performance among real estate agents. *J. Appl. Psychol.* 80 532–537. 10.1037/0021-9010.80.4.532

[B20] Crant J. M. (2000). Proactive behavior in organizations. *J. Manage.* 26 435–462. 10.1177/014920630002600304

[B21] Den Hartog D. N. Belschak F. D. (2012). When does transformational leadership enhance employee proactive behavior? The role of autonomy and role breadth self-efficacy. *J. Appl. Psychol.* 97 194–202. 10.1037/a0024903 21842977

[B22] Derue D. S. Nahrgang J. D. Wellman N. E. D. Humphrey S. E. (2011). Trait and behavioral theories of leadership: an integration and meta-analytic test of their relative validity. *Pers. Psychol.* 64 7–52. 10.1111/j.1744-6570.2010.01201

[B23] Dirks K. T. Ferrin D. L. (2001). The role of trust in organizational settings. *Organ. Sci.* 12 450–467. 10.1287/orsc.12.4.450.10640

[B24] Dysvik A. Kuvaas B. Buch R. (2016). Perceived investment in employee development and taking charge. *J. Manage. Psychol.* 31 50–60. 10.1108/JMP-04-2013-0117

[B25] Ertürk A. Vurgun L. (2015). Retention of IT professionals: examining the influence of empowerment, social exchange, and trust. *J. Bus. Res.* 68 34–46. 10.1016/j.jbusres.2014.05.010

[B26] Fiske S. Taylor S. (1984). *Social Cognition.* Reading, MA: Addison-Wesley.

[B27] Fong K. H. Snape E. (2015). Empowering leadership, psychological empowerment and employee outcomes: testing a multi-level mediating model. *Br. J. Manage.* 26 126–138. 10.1111/1467-8551.12048

[B28] Frese M. Fay D. (2001). Personal initiative: an active performance concept for work in the 21st century. *Res. Organ. Behav.* 23 133–187. 10.1016/S0191-3085(01)23005-6

[B29] Fritz C. Sonnentag S. (2009). Antecedents of day-level proactive behavior: a look at job stressors and positive affect during the workday. *J. Manage.* 35 94–111. 10.1177/0149206307308911

[B30] Fuller J. B. Marler L. E. Hester K. (2012). Bridge building within the province of proactivity. *J. Organ. Behav.* 33 1053–1070. 10.1002/job.1780

[B31] Griffin M. A. Parker S. K. Mason C. M. (2010). Leader vision and the development of adaptive and proactive performance: a longitudinal study. *J. Appl. Psychol.* 95 174–182. 10.1037/a0017263 20085414

[B32] Hassan S. Mahsud R. Yukl G. Prussia G. E. (2013). Ethical and empowering leadership and leader effectiveness. *J. Manage. Psychol.* 28 133–146. 10.1108/02683941311300252 22764828

[B33] He X. (2010). Why employee known but do not say: an indigenous empirical analysis base of employee silence. *Nankai. Bus. Rev.* 13 45–52. 10.3969/j.issn.1008-3448.2010.03.007

[B34] Hong Y. Liao H. Raub S. Han J. H. (2016). What it takes to get proactive: an integrative multilevel model of the antecedents of personal initiative. *J. Appl. Psychol.* 101 687–701. 10.1037/apl0000064 26653528

[B35] Hwang P. Han M. Chiu S. (2015). Role breadth self-efficacy and foci of proactive behavior: moderating role of collective, relational, and individual self-concept. *J. Psychol.* 149 1–20. 10.1080/00223980.2014.985284 25565604

[B36] Jonsson S. Muhonen T. Denti L. Chen K. (2015). Social climate and job control as mediators between empowering leadership and learning from a cross-cultural perspective. *Int. J. Cross Cult. Manage.* 15 1–15. 10.1177/1470595815572170

[B37] Kim S. Kim H. Lee J. (2015). Employee self-concepts, voluntary learning behavior, and perceived employability. *J. Manage. Psychol.* 30 264–279. 10.1108/JMP-01-2012-0010

[B38] Kramer R. M. (1999). Trust and distrust in organizations: emerging perspectives, enduring questions. *Annu. Rev. Psychol.* 50 569–598. 10.1146/annurev.psych.50.1.569 15012464

[B39] Lam C. F. Spreitzer G. Fritz C. (2014). Too much of a good thing: curvilinear effect of positive affect on proactive behaviors. *J. Organ. Behav.* 35 530–546. 10.1002/job.1906

[B40] Lee H. W. Pak J. Kim S. Li L. Z. (2016). Effects of human resource management systems on employee proactivity and group innovation. *J. Manag.* 10.1177/0149206316680029

[B41] Li M. Liu W. X. Zhang P. C. (2016). Linking empowering leadership and change-oriented organizational citizenship behavior. *J. Organ. Change Manag.* 29 732–750. 10.1108/JOCM-02-2015-0032

[B42] Li M. Zhang P. (2016). Stimulating learning by empowering leadership: can we achieve cross-level creativity simultaneously? *Leadersh. Organ. Dev. J.* 37 1168–1186. 10.1108/LODJ-01-2015-0007

[B43] Li N. Dan S. C. Kirkman B. L. (2017). Cross-level influences of empowering leadership on citizenship behavior: organizational support climate as a double-edged sword. *J. Manage.* 43 1076–1102. 10.1177/0149206314546193

[B44] Li R. Tian X. M. (2014). Supervisor authoritarian leadership and subordinate proactive behavior: test of a mediated-moderation model. *Acta. Psychol. Sin.* 46 1719–1733. 10.3724/SP.J.1041.2014.01719

[B45] Li S. L. He W. Kai C. Y. Long L. R. (2015). When and why empowering leadership increases followers’ taking charge: a multilevel examination in china. *Asia Pac. J. Manage.* 32 645–670. 10.1007/s10490-015-9424-1

[B46] López-Domínguez M. Enache M. Sallan J. M. Simo P. (2013). Transformational leadership as an antecedent of change-oriented organizational citizenship behavior. *J. Bus. Res.* 66 2147–2152. 10.1016/j.jbusres.2013.02.041

[B47] Lorinkova N. M. Pearsall M. J. Sims H. P. (2013). Examining the differential longitudinal performance of directive versus empowering leadership in teams. *Acad. Manage. J.* 56 573–596. 10.5465/amj.2011.0132

[B48] Martin S. L. Liao H. Campbell E. M. (2013). Directive versus empowering leadership: a field experiment comparing impacts on task proficiency and proactivity. *Acad. Manage. J.* 56 1372–1395. 10.5465/amj.2011.0113

[B49] Mayer R. C. Davis J. H. Schoorman F. D. (1995). An integrative model of organizational trust. *Acad. Manage. Rev.* 20 709–734. 10.5465/AMR.1995.9508080335

[B50] Mayer R. C. Gavin M. B. (2005). Trust in management and performance: who minds the shop while the employees watch the boss? *Acad. Manage. J.* 48 874–888. 10.5465/AMJ.2005.18803928

[B51] Nishii L. H. Wright P. M. (2008). “Variability within organizations: implications for strategic human resources management,” in *The People Make the Place: Dynamic Linkages Between Individuals and Organizations*, ed. Smith D. B. (Mahwah, NJ: Erlbaum), 225–248.

[B52] Nyhan R. C. Marlowe H. A. (1997). Development and psychometric properties of the organizational trust inventory. *Eval. Rev.* 21 614–635. 10.1177/0193841X9702100505

[B53] Ötken A. B. Cenkci T. (2012). The impact of paternalistic leadership on ethical climate: the moderating role of trust in leader. *J. Bus. Ethics* 108 525–536. 10.1007/s10551-011-1108-2

[B54] Ouyang K. Lam W. Wang W. (2015). Roles of gender and identification on abusive supervision and proactive behavior. *Asia Pac. J. Manage.* 32 1–21. 10.1007/s10490-015-9410-7

[B55] Parker S. K. (2000). From passive to proactive motivation: the impor- tance of flexible role orientations and role breadth self-efficacy. *Appl. Psychol.* 49 447–469. 10.1111/1464-0597.00025

[B56] Parker S. K. Bindl U. K. Strauss K. (2010). Making things happen: a model of proactive motivation. *J. Manage.* 36 827–856. 10.1177/0149206310363732 23379914

[B57] Parker S. K. Williams H. M. Turner N. (2006). Modeling the antecedents of proactive behavior at work. *J. Appl. Psychol.* 91 636–652. 10.1037/0021-9010.91.3.636 16737360

[B58] Pearce C. L. Sims H. P. Jr. (2002). Vertical versus shared leadership as predictors of the effectiveness of change management teams: an examinations of aversive, directive, transactional, transformational, and empowering leader behaviours. *Group Dyn. Theor. Res. Pract.* 6 172–197. 10.1037/1089-2699.6.2.172

[B59] Preacher K. J. Zyphur M. J. Zhang Z. (2010). A general multilevel SEM framework for assessing multilevel mediation. *Psychol. Methods* 15 209–233. 10.1037/a0020141 20822249

[B60] Saks A. M. Gruman J. A. Cooper-Thomas H. (2011). The neglected role of proactive behavior and outcomes in newcomer socialization. *J. Vocat. Behav.* 79 36–46. 10.1016/j.jvb.2010.12.007

[B61] Seibert S. E. Crant J. M. Kraimer M. L. (1999). Proactive personality and career success. *J. Appl. Psychol.* 84 416–427. 10.1037/0021-9010.84.3.416 10380421

[B62] Sharma P. N. Kirkman B. L. (2015). Leveraging leaders: a literature review and future lines of inquiry for empowering leadership research. *Group Organ. Manage.* 40 193–237. 10.1177/1059601115574906

[B63] Sonnentag S. (2003). Recovery, work engagement, and proactive behavior: a new look at the interface between nonwork and work. *J. Appl. Psychol.* 88 518–528. 10.1037/0021-9010.88.3.518 12814299

[B64] Spector P. E. (1985). Measurement of human service staff satisfaction: development of the Job Satisfaction Survey. *Am. J. Community Psychol.* 13 693–713. 10.1007/BF00929796 4083275

[B65] Spreitzer G. M. (1995). Psychological empowerment in the workplace: dimensions, measurement, and validation. *Acad. Manage. J.* 38 1442–1465. 10.2307/256865

[B66] Thomas J. P. Whitman D. S. Viswesvaran C. (2010). Employee proactivity in organizations: a comparative meta-analysis of emergent proactive constructs. *J. Occup. Organ. Psychol.* 83 275–300. 10.1348/096317910X502359

[B67] Tjosvold D. (1984). Effects of leader warmth and directiveness on subordinate performance on a subsequent task. *J. Appl. Psychol.* 69 422–427. 10.1037/0021-9010.69.3.422

[B68] Vecchio R. P. Justin J. E. Pearce C. L. (2010). Empowering leadership: an examination of mediating mechanisms within a hierarchical structure. *Leadersh. Q.* 21 530–542. 10.1016/j.leaqua.2010.03.014

[B69] Weseler D. Niessen C. (2016). How job crafting relates to task performance. *J. Manage. Psychol.* 31 672–685. 10.1108/JMP-09-2014-0269 25798721

[B70] Wu C. Parker S. K. (2017). The role of leader support in facilitating proactive work behavior: a perspective from attachment theory. *J. Manage.* 43 1025–1049. 10.1177/0149206314544745

[B71] Wu C. M. Chen T. J. (2015). Psychological contract fulfillment in the hotel workplace: empowering leadership, knowledge exchange, and service performance. *Int. J. Hosp. Manage.* 48 27–38. 10.1016/j.ijhm.2015.04.008

[B72] Wu M. Huang X. Li C. Liu W. (2012). Perceived interactional justice and trust-in-supervisor as mediators for paternalistic leadership. *Manage. Organ. Rev.* 8 97–121. 10.1111/j.1740-8784.2011.00283.x

[B73] Zhang X. Zhou J. (2014). Empowering leadership, uncertainty avoidance, trust, and employee creativity: interaction effects and a mediating mechanism. *Organ. Behav. Hum. Decis. Process.* 124 150–164. 10.1016/j.obhdp.2014.02.002

